# A joint time-frequency analysis of resting-state functional connectivity reveals novel patterns of connectivity shared between or unique to schizophrenia patients and healthy controls

**DOI:** 10.1016/j.nicl.2017.06.023

**Published:** 2017-06-17

**Authors:** Maziar Yaesoubi, Robyn L. Miller, Juan Bustillo, Kelvin O. Lim, Jatin Vaidya, Vince D. Calhoun

**Affiliations:** a The Mind Research Network, 1101 Yale Blvd NE, Albuquerque, NM 87106, USA; b Dept. of ECE, University of New Mexico, Albuquerque, NM 87131, USA; c Dept. of Psychiatry and Behavioral Science, University of New Mexico, Albuquerque, NM 87131, USA; d Dept. of Psychiatry, University of Minnesota, Minneapolis, MN 55414, USA; e Dept. of Psychiatry, University of Iowa, Iowa City, IA 52242, USA

**Keywords:** Resting-state functional connectivity, Dynamic and frequency-specific connectivity, Time-frequency analysis, Wavelet transform coherence

## Abstract

Functional connectivity of the resting-state (RS) brain is a vehicle to study brain dysconnectivity aspects of diseases such as schizophrenia and bipolar. Methods that are developed to measure functional connectivity are based on the underlying hypotheses regarding the actual nature of RS-connectivity including evidence of temporally dynamic versus static RS-connectivity and evidence of frequency-specific versus hemodynamically-driven connectivity over a wide frequency range. This study is derived by these observations of variation of RS-connectivity in temporal and frequency domains and evaluates such characteristics of RS-connectivity in clinical population and jointly in temporal and frequency domains (the *spectro-temporal domain*). We base this study on the hypothesis that by studying functional connectivity of schizophrenia patients and comparing it to the one of healthy controls in the spectro-temporal domain we might be able to make new observations regarding the differences and similarities between diseased and healthy brain connectivity and such observations could be obscured by studies which investigate such characteristics separately.

Interestingly, our results include, but are not limited to, a spectrally localized (mostly mid-range frequencies) modular dynamic connectivity pattern in which sensory motor networks are anti-correlated with visual, auditory and sub-cortical networks in schizophrenia, as well as evidence of lagged dependence between default-mode and sensory networks in schizophrenia. These observations are unique to the proposed augmented domain of connectivity analysis. We conclude this study by arguing not only resting-state connectivity has structured spectro-temporal variability, but also that studying properties of connectivity in this joint domain reveals distinctive group-based differences and similarities between clinical and healthy populations.

## Highlights

• RS-connectivity of Schizophrenia and healthy is studied in domain of time and frequency.
• Dynamic connectivity is now superposition of connectivities with unique frequencies.
• Augmented domain reveals novel connectivity patterns unique to schizophrenia/healthy.

## 1 Introduction

Schizophrenia is a complex psychiatric illness with estimated occurrence rate of 1% (Bhugra, 2005) in global population. The main objective of early studies of this disorder had been definition and diagnosis via symptoms, later moving toward a search for biomarkers rather than relying exclusively on observed or self-reported symptomology (Keshavan et al., 2013). Although there is additional complexity emerging regarding the validity of current state of classification and diagnostic criteria for complex mental disorders (Cheniaux et al., 2008, Kotov et al., 2013) we cannot ignore the power of biological markers from genomics and brain imaging in refining diagnostic criteria.

Functional magnetic resonance imaging (fMRI), as a non-invasive method to capture hemodynamic mediated activity of brain regions due to the function of the brain at the given time, is an appealing tool for studying schizophrenia; a brain disorder that leads to disturbances of thought, cognition and emotion (Schultz and Andreasen, 1999, Andreasen and Flaum, 1991). Functional connectivity with fMRI has been widely used to study schizophrenia (Garrity, 2007, Calhoun et al., 2011) due to the fact that the disease is recognized as a dysconnectivity disorder (Pettersson-Yeo et al., 2011, Fornito et al., 2012, Friston, 1998).

Evidence of anomalies in brain connectivity of patients goes back to early studies (Wernicke, 1906) in which psychosis was associated with disruption of association fiber tracts in the brain. Since then, extensive work in identifying changes in structural and functional connectivity has been performed. Between the two, functional analyses has advantages which allows to view the “living” brain while it performs experimental or internally-driven tasks.

Functional connectivity during the resting-state, compared to task-based designs, is shown to span a broader range of frequencies in neurophysiological activation (Xiong et al., 1999) as well as engaging more functional networks(Smith et al., 2009, Damaraju et al., 2014a, Gusnard et al., 2001). Identified networks include default mode (DM) networks, which have been associated with self-reflection and self-monitoring (Flashman et al., 2004) and networks connected with auditory hallucination and paranoid ideation (Friston and Frith, 1995).

Observations of resting-state functional connectivity of schizophrenia include both increased/hyper and decreased/hypo connectivity compared to healthy-controls between various brain regions, though predominantly the latter. For example there are many studies have reported significant weaker whole-brain connectivity in schizophrenia patients compared to health controls(Argyelan et al., 2014; Lynall et al., 2010) although hyper-connectivity is also observed in-between default mode networks and between default-mode and networks associated to cognitive demands appearing as increasing anti-correlation(Zhou et al., 2007). However, results are not always consistent between studies. For example, both hyper and hypo connectivity have been reported between DM networks in schizophrenia (Fornito et al., 2012; Whitfield-Gabrieli et al., 2009). Such differences in findings can be due to uncontrolled sources of variations among subjects, which may affect the final estimation of connectivity (such as motion artefact as one of extensively studied artefact (Van Dijk et al., 2012), or can be due to the fact than the chosen method for the estimation of connectivity is not capturing all aspects of brain connectivity. An example for the latter is transition from temporally stationary estimation of connectivity toward a temporally dynamic connectivity based on overwhelming evidences that whole-brain resting-state connectivity is in fact temporally dynamic(Calhoun et al., 2014). Sliding-window approaches have been the most commonly used to capture such time-varying connectivity(Allen et al., 2014) also it has been used to study schizophrenia in the context of transient states of connectivity (Damaraju et al., 2014a) extending observation of static hypo-connectivity in schizophrenia to spending more time in a hypo-connected state.

Common studies of dynamic functional connectivity have been focused on the dynamics of the degree of dependence by measuring co-activation, which is limited to observing the changes between positive, negative or weak co-activation. However, based on recent studies of frequency profiles of functional connectivity, evidence of dynamics along the frequency dimension have also been shown. This includes observing differences in the frequency of activation and co-activation between different regions or functional networks of the brain(Calhoun et al., 2011; Yu et al., 2014; Meda et al., 2015; Hoptman et al., 2010) and also, more recently, it includes observation in the temporal dynamic changes of the frequency of co-activation between the same pair of regions or networks(Chang and Glover, 2010; Yaesoubi et al., 2015). The former observations have shown that evaluations of frequency specific activation and co-activation enable us to capture significant differences between groups of patients and healthy controls (Miller et al., 2016) as it is shown to carry useful information related to the underlying neurophysiological processes. For example, the default-mode network has been shown to exhibit significantly more high frequency fluctuations in patients and significantly less low frequency fluctuations in controls, perhaps related to decreased cognitive efficiency (Garrity, 2007).

A common hypothesis derived from both groups of observations is that observed activation in a given brain region may originate from various neurophysiological sources of fluctuations with unique spectral properties (Penttonen, 2003; Buzsaki and Draguhn, 2004) which also extends to the frequency variation in the measured co-activation. Here, motivated by both groups of studies, we leverage a more general framework to simultaneously investigating temporally dynamic and frequency-specific functional connectivity during resting-state fMRI.

Our proposed joint analysis has been enabled by recent studies that have developed methods to simultaneously capture temporal behavior as well as frequency and phase profiles associated with each state. Such studies leverage a time-frequency decomposition to capture functional connectivity between a few selected ROIs in the joint domains (Chang and Glover, 2010) (Yaesoubi et al., 2015). An important advantage of the proposed framework is that the connectivity state of the brain at given time-point can be studied as a superposition of multiple frequency-specific connectivity states. Additionally, the phase information encoded in the frequency domain enables us to capture a delayed correlation; similar in concept to a study that identifies delayed correlation between relatively shifted time-courses among multiple brain networks (Jafri et al., 2008).

In this work we investigated the above proposed pipeline (Jafri et al., 2008) for capturing the spectro-temporal whole-brain connectivity patterns characterized jointly by time, frequency and phase (lagged dependence) in healthy controls and schizophrenia patients. We show that such decomposition enables us to observe significantly different characterization of connectivity between the two groups. We also observe that when transient connectivity states are defined at the group level, shared between both groups, occupancy rates and dwell times for the group-level differ significantly between patients and controls.

## 2 Materials and method

### 2.1 Participants, image acquisition and pre-processing

Subjects include 163 healthy controls (46 females) with average age of 36.9 and 151 (37 females) patients diagnosed with schizophrenia with average age of 37.8. In accordance with the internal review boards of corresponding institutes, informed consent was obtained from all the subjects. Details on the image acquisition and the pre-processing are provided in Supplementary Material A.

#### 2.1.1 Group-ICA and post-processing

We study connectivity between anatomically and functionally meaningful regions in the brain. We choose group spatial independent component analysis (gsICA) as the data-driven approach to define these regions with no need for prior knowledge of the regions or a task-design. It is achieved by linearly decomposing voxel-level time series into maximally independent spatial maps with corresponding time-courses per-subject (Calhoun et al., 2001, Calhoun and Adali, 2012). The GIFT toolbox implementation of gsICA is used. More details on implementation of gsICA in GIFT are provided in Supplementary Material B.

### 2.2 Temporally-dynamic and frequency-specific connectivity states

To estimate connectivity, in the spectro-temporal domain, we first, decompose each subject-specific network time-courses into a time-frequency domain by leveraging a wavelet decomposition by convolving each time-course with complex Morlet wavelet as our choice of wavelet kernel. The complex Morlet kernel has the following formulation, which has both real and imaginary parts:

(1) $$\frac{1}{\sqrt{2\pi }\sigma }e^{2\pi {\mathrm{if}}_{c}t}\times e^{-t^{2}/2\sigma^{2}}$$

The kernel has a Gaussian-shaped frequency spectrum whose *f*_*c*_ is center frequency, and *σ* is standard deviation. *σ* is set to 0.02 Hz throughout the study.

**Fig. 1:**
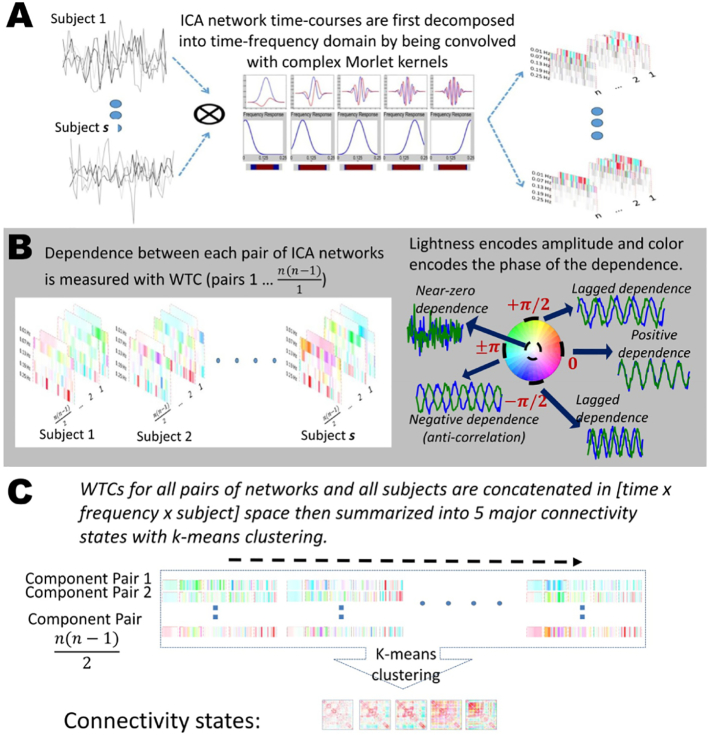
Pipeline to capture connectivity states in a joint time and frequency domain.

Each input time-course is convolved with 5 different Morlet kernels each centered equally in the interval of 0.01 and 0.25 Hz which when stacked, it would result into the time-frequency representation of the input time-course (Fig. 1A).

Next we use wavelet transform coherence (WTC) for the estimation of dependence in this joint domain. WTC is defined as follows:

(2) $$R=\frac{S\left(W^{\mathrm{xy}}\right)}{\sqrt{S^{'}\left({\left|W^{x}\right|}^{2}\right)}\sqrt{S^{'}\left({\left|W^{y}\right|}^{2}\right)}}$$

W^xy^ represents element-wise conjugate multiplication of wavelet transforms of input signals x and y which are represented as W^x^ and W^y^. S and S^′^ are the smoothing parameters. Details on the WTC and its formulation are provided in Yaesoubi et al. (Yaesoubi et al., 2015).

WTC is used to measure dependence between all pairs of time-frequency representation of network time-courses. Such dependence is what we call “dynamic coherence”. Dynamic coherence has a complex value with both real and imaginary parts. Its magnitude measures the degree of the dependence within a given frequency band between two time-series at a given point in time, with phase capturing the lag at which maximum correlation is achieved.

Fig. 1B Right, explains this property as well as the color-coding that is used through out of this work to present our complexed-value dynamic coherence. The lightness of the colormap represents the magnitude of the measurement and phase is encoded with the selected circular colormap.

Following procedure proposed by Yaesoubi et al.(Yaesoubi et al., 2015) to estimate spectro-temporal connectivity states, estimations of dynamic coherence for all pairs of networks are concatenated along time, frequency and subjects followed by a clustering analysis to summarize it into finite number of states. We use k-means clustering and *k* is set to ‘5’ (Fig. 1C).

## 3 Results

The results in the paper are organized into two sets. In the first set, we estimate spectro-temporal connectivity states in the time-frequency domain separately for healthy controls and patients. This allows us to inspect differences in the connectivity patterns and frequency and phase profiles of the states between patients and controls. In the second set, we estimate connectivity states shared among all subjects. This allows us to investigate differences between the two groups with respect to their occupancy rates (amount of time subjects live at a specific state during the course of the scan) as well as their tendency to stay in each state.

**Fig. 2:**
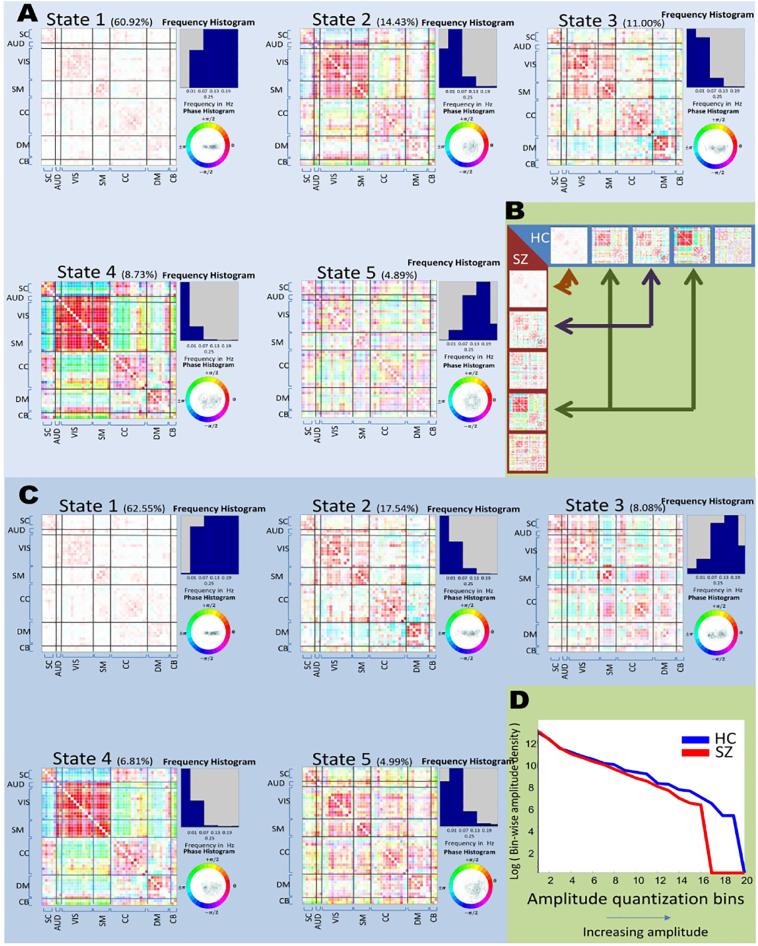
(A) Connectivity states of healthy controls defined in the time-frequency domain and similarly in (C) for schizophrenia patients. A detailed description of the networks on the rows and columns of the each state is provided in Appendix B Fig. S1. (B) Maximally correlated connectivity states between SZs and HCs. (D) Plot showing overall stronger connectivity in HCs compared to SZs.

In Fig. 2A,C we show group-specific (healthy controls and schizophrenia patients) spectro-temporal connectivity states along with the corresponding phase and frequency. States are sorted based on their occurrence rates during the course of the scan.

Correlation between connectivity patterns of pair of states each belonging to a different group of subjects (Fig. 2B) shows that most commonly occurring state (state 1) is shared among the groups and has a high frequency range (frequency profiles being left-skewed peaking at maximum possible frequency of 0.25 Hz). HC state 3 has maximum correlation (*r* = 0.8856) with SZ state 2, and states share similar frequency profiles. Finally, the connectivity pattern of SZ state 4 maps to both of the HC states 2 and 4 (*r* = 0.7971, 0.9038 respectively); however, the frequency profile of SZ state 4 only matches the frequency profile of HC state 4 (Fig. 2B). Furthermore, SZ state 3 and HC state 5 share similar frequency profiles, but both have unique connectivity patterns which are minimally correlated with any other states of each group. Note that many of these states are only identifiable in the joint time-frequency domain. If temporally dynamic whole-brain connectivity states were estimated on full-spectrum (Damaraju et al., 2014a, Allen et al., 2014), states with different frequency profiles might have been merged and the patterns would have been blurred across states. This would also be true if whole-brain connectivity was estimated on full time-courses as in coherence analysis. For example, SZ state 3 with a unique connectivity patterns, specifically a pronounced anti-correlation between somatomotor and visual/auditory/sub-cortical networks would not have been distinguished from state 1 due to the similar frequency profile.

We also investigated differences between amplitude and phase of the dynamic coherence of the connectivity states that are maximally correlated between HCs and SZs. This enables us to compare connectivity level of each group at each state as a notion of relative hyper or hypo connectivity. The details on how we performed this contrast analysis is provided in Supplementary Material C but in summary, we estimate the degree of difference between median of network-pairs' dynamic coherence (performed separately for amplitude and phase) of each state between the two groups.

**Fig. 3:**
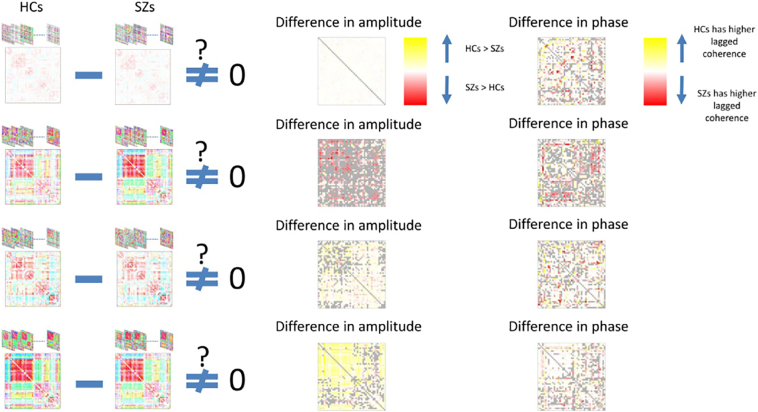
Identification of component pairs with significant differences in either amplitude or phase of the dynamic coherence between maximally correlated states. Column 2 shows SZ states which are maximally correlated to the HC states on column 1. Column 3 shows difference in amplitude of component-pair dynamic coherence between HC and SZ which reject the null hypothesis. Gray entries show the ones which did not reject the null. Column 4 shows difference in phase with similar analysis.

Fig. 3 summarizes this analysis between maximally correlated states (Fig. 2B). Yellow means HCs have significantly higher median of amplitude of dynamic coherence in the corresponding network pair and red means the reverse (second column). Same color map is used for difference in the phase (third column). Gray also means no significant differences. We clearly see that both phase and amplitude contribute in the difference between HCs and SZs for given component pairs.

Moreover, we investigated the correlation of age and gender of subjects of each group and including symptom scores (positive, negative and general psychopathology scales) and medication score (chlorpromazine equivalency (CPZ) scores) of only patient group to the estimate to the average of connectivity corresponding to each state of each subject. The analysis was performed very similarly as the above analysis (similarly it was performed separately for amplitude and phase of the averaged connectivity). However we did not observe any strong effect of age, gender, symptom or CPZ scores to either phase or amplitude of subject-wise and per-state averaged connectivity.

**Fig. 4:**
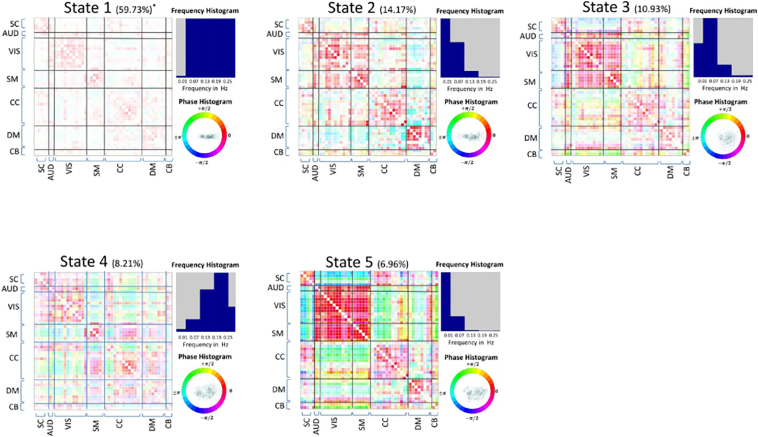
Connectivity states defined in time-frequency domain over all subjects regardless of the diagnosis.

In second set of the results, we investigate differences between groups by studying the occupancy rates and tendency to stay in a given state (dwell time) for patients and controls. We perform k-means clustering on all the subjects (regardless of the diagnosis) and identify 5 spectro-temporal connectivity states as in Fig. 4 accompanied by phase and frequency profiles. There are evident similarities between these states and the connectivity patterns in Fig. 3, Fig. 4.

**Fig. 5:**
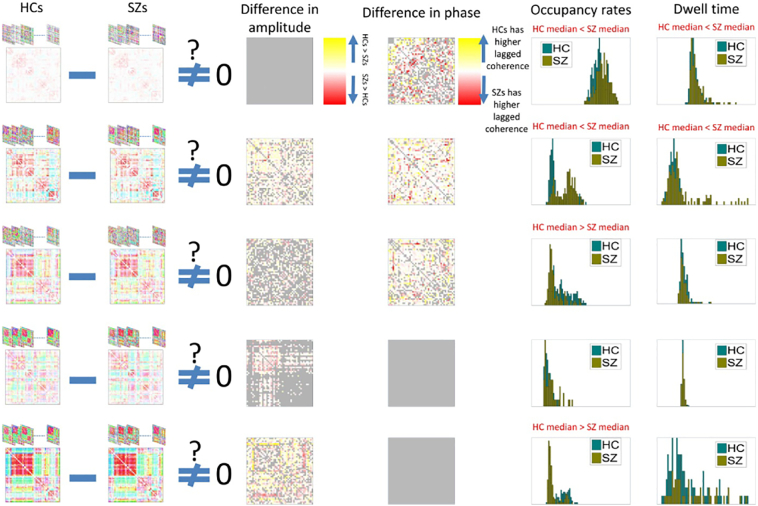
Differences in amplitude or phase of dynamic coherence of components belonging to the same state but different group. Column 3: difference in amplitude, column 4: difference in the phase of the dynamic coherence. Column 5: histogram of occupancy measure of HCs and SZs subjects, column 6: histogram of dwell times.

Next, we measure occupancy rates of each subject in each state by counting the number of time-frequency points which were assigned to a given state during the scan. This is followed by group difference analysis on the distribution of occupancy rates between HCs and SZs. Interestingly, in all states except state 4, we observed significant differences (FDDR adjusted *p*-value < 0.01) between the two groups (Kolmogorov-Smirnov test for difference in medians). Schizophrenia patients were more likely to occupy state 1 (low global coherence, higher frequency profile) and state 2 (negative DMN-to-other coherence, otherwise high global coherence with relatively lower frequency profile). On the other hand, healthy controls had a greater tendency to occupy state 3 (high coherence between sensory networks and negative coherence between subcortical and sensory networks and also diminished DMN-to-other coherence with low frequency profile) and state 5 (extremely modularized coherence structure, very high intra-domain coherence for all domains plus high subcortical-to-DMN, cognitive control and cerebellum, and very low frequency profile). Group-wise distribution of occupancy rates is represented in column 5 of Fig. 5. We also assessed the tendency of subjects to linger in a given state by counting number of consecutive occurrences of each state (this is done separately for each frequency band). We then take the median of these measurements for each subject as our measure of state-specific dwell time. As with the occupancy rate, we find patients have significantly higher dwell-times in states 1 and 2 as shown in column 6 of Fig. 7. As before, we regressed out variation due to the other subject-variables (age, gender, site and motion parameters) from both measures before conducting these tests.

Similar to the results in Fig. 3 differences of amplitude and phase of dynamic coherence between HCs and SZs is analyzed as represented on column 3 (differences in amplitude) and column 4 (difference in phase) of Fig. 5.

## 4 Discussion

In this study, we investigated whole-brain resting-state connectivity differences between schizophrenia patients and healthy controls in a framework that smoothly integrates frequency domain characteristics with temporal dynamics of connectivity, phenomena that have previously been explored separately, but not combined.

When we separately estimated spectro-temporal connectivity states for patients and controls, we identified connectivity states shared by both groups as well as connectivity states unique to each group. An interesting observation based on this result is that the most similar connectivity states between the patient and control groups involve state-pairs that either both have very high frequency profiles (HC state 1 and SZ state 1) or low frequency profiles (States 2 and 4 of HCs and state 4 of SZs). Consequently, most of the group differences occur in connectivity states with relatively middle range frequency profiles, e.g. HC state 5 whose a frequency profile peaks at around ~ 0.17 Hz and is minimally correlated with any SZ states, and also SZ states 3 and 5, which together cover a range of middle frequencies between 0.07 Hz to 0.17 Hz. There is recognizable unique modularity in these states. For example, in SZ state 2 we clearly observe relatively strong and positive correlation between all the subcortical (SC), auditory (AUD), and visual (VIS) networks. At the same time, however, uniquely among all of the SZ and HC-specific states, SZ state 2 features negative correlations between somatomotor (SM) networks and the SC, AUD and VIS networks. To the best of our knowledge, this is the first evidence for this particular pattern of connectivity in schizophrenia patients, and it is only identifiable when connectivity is analyzed jointly in time and frequency. Among the HC states, state 5 exhibits the most similar modular patterning to SZ state 2 but with a different connectivity pattern between SC and AUD/VIS networks (anti-correlated in HC state 5, positively correlated in SZ state 2) and between SM and AUD/VIS (positively correlated in HC state 5, anti-correlated in SZ state2). Previous studies (Woodward et al., 2012, Anticevic et al., 2014) have reported hyper-connectivity between the thalamic and sensory networks in schizophrenia patients, which here appears as a positive connectivity between all subcortical and sensory networks compared to negative connectivity in HCs between the same networks. Again such modularity only exists in the joint domain since: first, this modularity occurs in states with a unique frequency profiles (having a mid to high frequency range) and could not be captured when states were estimated over all frequencies (as is the case in conventional dynamic connectivity studies); and second, it is different from some other states with which it has an overlapping frequency profile. In fact, if we had studied connectivity only along frequency dimension, states 1 and 2 of SZ would have blurred along temporal domain and we were unable to observe such pronounced modularity unique to SZ.

Another observation is that HC states tend to have more dispersion with regard to phase of dynamic coherence representing lagged dependence rather than clearly positive or negative (anti) correlation. This can be observed from the phase/amplitude histograms of states 2, 4 and 5 of HCs but is only seen in states 4 and 5 of SZs.

Moreover, we observe that HC states have an overall stronger connectivity compared to SZs.

For this, we first estimate the amplitude distribution of each state as well as the subject-wise occupancy rates of the states. Then, from these two, we estimate subject-wise amplitude distribution uniformly quantized in 20 bins covering a range of 0 to the maximum amplitude of all states. Fig. 2D shows log of the median of these distributions at each bin separately for HCs and SZs and we observe as amplitude increases, although the log of the median of both groups decreases, for SZs it decay faster. This observation is in line with studies reporting decreased connectivity of SZs between wider range of networks or ROIs (Bluhm et al., 2007, Liang et al., 2006, Meda et al., 2012).

Meanwhile, although HCs showed an overall stronger connectivity than SZs, the dynamic nature of connectivity does not necessarily follow this overall observation. By revisiting rows 2 and 4 of Fig. 3, we observe although SZ state 4 has maximum correlation to both states 2 and 4 of HCs, the directionality of the difference in amplitudes changes when SZ state 4 and HC state 2 are compared versus when SZ state 4 and HC state 4 are compared (SZ > HC coded as red, HC > SZ is coded as yellow). This shows that HCs experience both higher and lower amplitude of dynamic coherence in similar connectivity patterns but in different states (2 and 4) in comparison to SZs having less variation in amplitude in a similar state (only in state 4).

In the second set of the results, by following Damaraju et al. (Damaraju et al., 2014b), we explicitly compare differences in the dynamic behavior of the subjects with respect to occupancy rates of each state as well as tendency to stay in a given state for a period of time (dwell time). Note that in this set, states are shared among all subjects. Our analysis shows significant differences between the groups based on both measurements. Core observations from this set of results is that first, we see that patients have higher occupancy rates and longer dwell times in state 1, a hypoconnected state associated with mid and high range frequency profile. The significant association of patients with this state pulls together two disparate and consistent findings from previous studies: First, patient network time-courses have more high frequency content than controls (Turner et al., 2013, Calhoun et al., 2008) and second, patients' networks tend to be more weakly (hypo) connected than those of controls(Bluhm et al., 2007, Liang et al., 2006, Meda et al., 2012). We also see that healthy and patients have different occupancy rates in states 2 and 5 which are observed at the lowest frequencies and exhibiting similar modular patterning – except for the valency of subcortical connections to Aud/Vis/SM (negative for state 5, positive for state 2). State 5 with a narrower frequency profile and a sharper modularization and a stronger connections between SC and Aud/Vis/SM, as compared to state 2, is more occupied by HCs while state 2 is more occupied by SZs. Similarly, state 3 with a mid-range frequencies profile, is more occupied by HCs. State 3 looks like a washed out version of state 5 but having a broader frequency profile while sharing the negative SC-to-Aud/Vis/SM connectivity which however is more sharply exhibited by state 5. We can interpret these group differences as extensions of previously reported results on schizophrenia and both dysconnectivity and altered frequency-domain characteristics (Whitfield-Gabrieli et al., 2009, Liang et al., 2006, Damaraju et al., 2014b) to the spectro-temporal expansion of connectivity.

Furthermore, consistent with the contrast analysis of phase and amplitude for the group-specific connectivity states of the first set of the results (Fig. 3), we also observe amplitude and phase differences between patients and controls in states from the second set of results. The clustering and states were drawn from the entire population, but clearly are not only occupied differentially by patients and controls, but also within a given cluster the observations from patients and controls are exhibiting significant differences in their phase and amplitude properties.

## 5 Limitations and future works

There are limitations related to both theoretical aspects of dynamic coherence and its implementation in this work. Dynamic coherence is not able to measure dependence across frequency consequently, we are not able to study cross-frequency connectivity of the brain. Possible future work would be investigating cross-frequency connectivity in the similar framework by possibly leveraging the phase information of dynamic coherence similar to studies of phase-synchronization.

Next, for summarization of dynamic coherence estimations into finite number of states, we used k-means analysis. Although our observation was that the captured connectivity states, represented by centroids of the k-means clusters, explain most of the variation of the dynamic coherence among subjects, we believe there is still room for investigating other summarization approached with different underlying assumptions which might complement the given states.

## 6 Conclusion

In this work, resting-state connectivity is studied in a joint domain of time and frequency. Our results provide strong evidence for systematic variation of connectivity that is characterized jointly in both domains, and also reveal novel differences and similarities between diseased and healthy subjects. The observations are unique to connectivity characterized jointly in the time and frequency domain and c thus have been obscured in previous studies of resting-state connectivity and schizophrenia.

## Appendix A Supplementary material

In this file you can access supplementary material which were referred from the main text. Image 1

## References

[bb0015] Allen E.A. (2014). Tracking whole-brain connectivity dynamics in the resting state. Cereb. Cortex.

[bb0020] Andreasen N.C., Flaum M. (1991). Schizophrenia: the characteristic symptoms. Schizophr. Bull..

[bb0025] Anticevic A. (2014). Characterizing thalamo-cortical disturbances in schizophrenia and bipolar illness. Cereb. Cortex.

[bb0030] Argyelan M. (2014). Resting-state fMRI connectivity impairment in schizophrenia and bipolar disorder. Schizophr. Bull..

[bb0035] Bhugra D. (2005). The global prevalence of schizophrenia. PLoS Med..

[bb0040] Bluhm R.L. (2007). Spontaneous low-frequency fluctuations in the BOLD signal in schizophrenic patients: anomalies in the default network. Schizophr. Bull..

[bb0045] Buzsaki G., Draguhn A. (2004). Neuronal oscillations in cortical networks. Science.

[bb0050] Calhoun V.D., Adali T. (2012). Multisubject independent component analysis of fMRI: a decade of intrinsic networks, default mode, and neurodiagnostic discovery. IEEE Rev. Biomed. Eng..

[bb0055] Calhoun V.D. (2001). A method for making group inferences from functional MRI data using independent component analysis. Hum. Brain Mapp..

[bb0060] Calhoun V.D., Kiehl K.A., Pearlson G.D. (2008). Modulation of temporally coherent brain networks estimated using ICA at rest and during cognitive tasks. Hum. Brain Mapp..

[bb0065] Calhoun V.D. (2011). Exploring the psychosis functional connectome: aberrant intrinsic networks in schizophrenia and bipolar disorder. Front. Psych..

[bb0070] Calhoun Vince D. (2014). The chronnectome: time-varying connectivity networks as the next frontier in fMRI data discovery. Neuron.

[bb0075] Chang C., Glover G.H. (2010). Time-frequency dynamics of resting-state brain connectivity measured with fMRI. NeuroImage.

[bb0080] Cheniaux E. (2008). Does schizoaffective disorder really exist? A systematic review of the studies that compared schizoaffective disorder with schizophrenia or mood disorders. J. Affect. Disord..

[bb0085] Damaraju E. (2014). Dynamic functional connectivity analysis reveals transient states of dysconnectivity in schizophrenia. Neuroimage Clin..

[bb0090] Damaraju E. (2014). Dynamic functional connectivity analysis reveals transient states of dysconnectivity in schizophrenia. Neuroimage Clin..

[bb0100] Flashman L.A., Beitman B.D., Nair J. (2004). Disorders of insight, self-awareness, and attribution in schizophrenia. Self-Awareness Deficits in Psychiatric Patients: Neurobiology, Assessment, and Treatment.

[bb0105] Fornito A. (2012). Schizophrenia, neuroimaging and connectomics. NeuroImage.

[bb0110] Friston K.J. (1998). The disconnection hypothesis. Schizophr. Res..

[bb0115] Friston K.J., Frith C.D. (1995). Schizophrenia: a disconnection syndrome?. Clin. Neurosci..

[bb0120] Garrity (2007). Aberrant 'default mode' functional connectivity in schizophrenia. Am. J. Psychiatry.

[bb0125] Gusnard D.A. (2001). Medial prefrontal cortex and self-referential mental activity: relation to a default mode of brain function. Proc. Natl. Acad. Sci. U. S. A..

[bb0130] Hoptman M.J. (2010). Amplitude of low-frequency oscillations in schizophrenia: a resting state fMRI study. Schizophr. Res..

[bb0135] Jafri M.J. (2008). A method for functional network connectivity among spatially independent resting-state components in schizophrenia. NeuroImage.

[bb0140] Keshavan M.S. (2013). Reimagining psychoses: an agnostic approach to diagnosis. Schizophr. Res..

[bb0145] Kotov R. (2013). Boundaries of schizoaffective disorder revisiting Kraepelin. JAMA Psychiatry.

[bb0150] Liang M. (2006). Widespread functional disconnectivity in schizophrenia with resting-state functional magnetic resonance imaging. Neuroreport.

[bb0155] Lynall M.E. (2010). Functional connectivity and brain networks in schizophrenia. J. Neurosci..

[bb0160] Meda S.A. (2012). Differences in resting-state functional magnetic resonance imaging functional network connectivity between schizophrenia and psychotic bipolar probands and their unaffected first-degree relatives. Biol. Psychiatry.

[bb0165] Meda S.A. (2015). Frequency-specific neural signatures of spontaneous low-frequency resting state fluctuations in psychosis: evidence from bipolar-schizophrenia network on intermediate phenotypes (B-SNIP) consortium. Schizophr. Bull..

[bb0170] Miller R.L., Yaesoubi M., Calhoun V.D. (2016). Cross-frequency rs-fMRI network connectivity patterns manifest differently for schizophrenia patients and healthy controls. IEEE Signal Process. Lett..

[bb0175] Penttonen M. (2003). Natural logarithmic relationship between brain oscillators. Thalamus Relat. Syst..

[bb0180] Pettersson-Yeo W. (2011). Dysconnectivity in schizophrenia: where are we now?. Neurosci. Biobehav. Rev..

[bb0185] Schultz S.K., Andreasen N.C. (1999). Schizophrenia. Lancet.

[bb0190] Smith S.M. (2009). Correspondence of the brain's functional architecture during activation and rest. Proc. Natl. Acad. Sci. U. S. A..

[bb0195] Turner J.A. (2013). A multi-site resting state fMRI study on the amplitude of low frequency fluctuations in schizophrenia. Front. Neurosci..

[bb0200] Van Dijk K.R.A., Sabuncu M.R., Buckner R.L. (2012). The influence of head motion on intrinsic functional connectivity MRI. NeuroImage.

[bb0205] Wernicke C. (1906). Grundriss der Psychiatrie in klinischen Vorlesungen.

[bb0210] Whitfield-Gabrieli S. (2009). Hyperactivity and hyperconnectivity of the default network in schizophrenia and in first-degree relatives of persons with schizophrenia. Proc. Natl. Acad. Sci. U. S. A..

[bb0215] Woodward N.D., Karbasforoushan H., Heckers S. (2012). Thalamocortical dysconnectivity in schizophrenia. Am. J. Psychiatry.

[bb0220] Xiong J.H. (1999). Interregional connectivity to primary motor cortex revealed using MRI resting state images. Hum. Brain Mapp..

[bb0225] Yaesoubi M. (2015). Dynamic coherence analysis of resting fMRI data to jointly capture state-based phase, frequency, and time-domain information. NeuroImage.

[bb0230] Yu R. (2014). Frequency-specific alternations in the amplitude of low-frequency fluctuations in schizophrenia. Hum. Brain Mapp..

[bb0235] Zhou Y. (2007). Functional disintegration in paranoid schizophrenia using resting-state fMRI. Schizophr. Res..

