# Supplementary material for: A joint time-frequency analysis of resting-state functional connectivity reveals novel patterns of connectivity shared between or unique to schizophrenia patients and healthy controls
Source: Neuroimage Clin. 2017 Jun 17;15:761–8. doi: 10.1016/j.nicl.2017.06.023 (PMC5496209; doi:10.1016/j.nicl.2017.06.023)
Supplement: Supplementary file 1 — In this file you can access supplementary material which were referred from the main text. [file mmc1.docx]

# Supplementary material

# A

| Site/3T Scanner | Num. of subjects | Num. of HC | Num. of Sz | Age mean ± std dev | | SZ Median SES Education | HC Median SES Education |
| --- | --- | --- | --- | --- | --- | --- | --- |
|  |  |  |  | SZ | HC |  |  |
| Duke/  GE | 52 | 28  (7 F) | 24  (4 F) | 34.5 ± 8.4 | 34.1 ± 9.25 | 3 | 2 |
| Iowa/  Siemens | 18 | 9  (3 F) | 9  (2 F) | 41.1 ± 12.5 | 39.3 ± 9.07 | 4 | 3 |
| UCI/ Siemens | 54 | 28  (6 F) | 26  (5 F) | 44.1 ± 11.9 | 43.1 ± 12.7 | 4 | 2 |
| UCLA/ Siemens | 51 | 28  (5 F) | 23  (5 F) | 34.9 ± 11.9 | 35.6 ± 11.6 | 3 | 3 |
| UCSF/ Siemens | 27 | 14  (3 F) | 13  (4 F) | 36.7 ± 10.7 | 36.4 ± 9.1 | 3 | 2 |
| UMN/ Siemens | 57 | 28  (7 F) | 29  (5 F) | 36.3 ± 11.1 | 34.6 ± 10.7 | 3 | 2 |
| UNM/ Siemens | 55 | 28  (4 F) | 27  (7 F) | 38.6 ± 11.7 | 36.8 ± 10.4 | 3 | 2 |

Table S 1: Subject demographic information.

### Details of image acquisition and pre-processing

#### Image Acquisition

fMRI scans of the subjects during a resting-state with eyes closed, were acquired across 7 different sites. Table S1 summarizes demographic information of the subjects. Diagnosis of the patients was based on the structured clinical interview for DSM-IV-TR axis I disorders (SCID-I/P). Illness duration of all the patients was at least one year. Medication information of the patients is provided in Figure S 1A. We used chlorpromazine equivalent scores in throughout our analysis and the histogram of the scores is provided in Figure S 1A Right. Distribution of patients’ positive, negative and general psychopathology scales are provided in Figure S 1B.

Scans for each subjects constitute 162 volumes of T_2_^*^-weighted functional images with EPI sequence. Same scanners a 3T Siemens Tim Trio System were used in 6 sites and a 3T General Electric Discovery MR750 scanner was used in only one of the sites. All of the scans had FOV = 11x220 mm, matrix size = 64 x 64, TR = 2 s, TE = 30 ms and FA = 770.

#### Pre-processing

Head motion, slice-timing correction and spatial normalization to the Montreal Neurological Institute (MNI) template was applied to the data using SPM toolbox followed by despiking using AFNI to remove outliers. The BlurToFWHM algorithm as implemented in AFNIs was used for spatial smoothing of the data and lastly all voxel-wise time series were z-scored to normalize the variance before performing group spatial independence component analysis (gsICA).

###
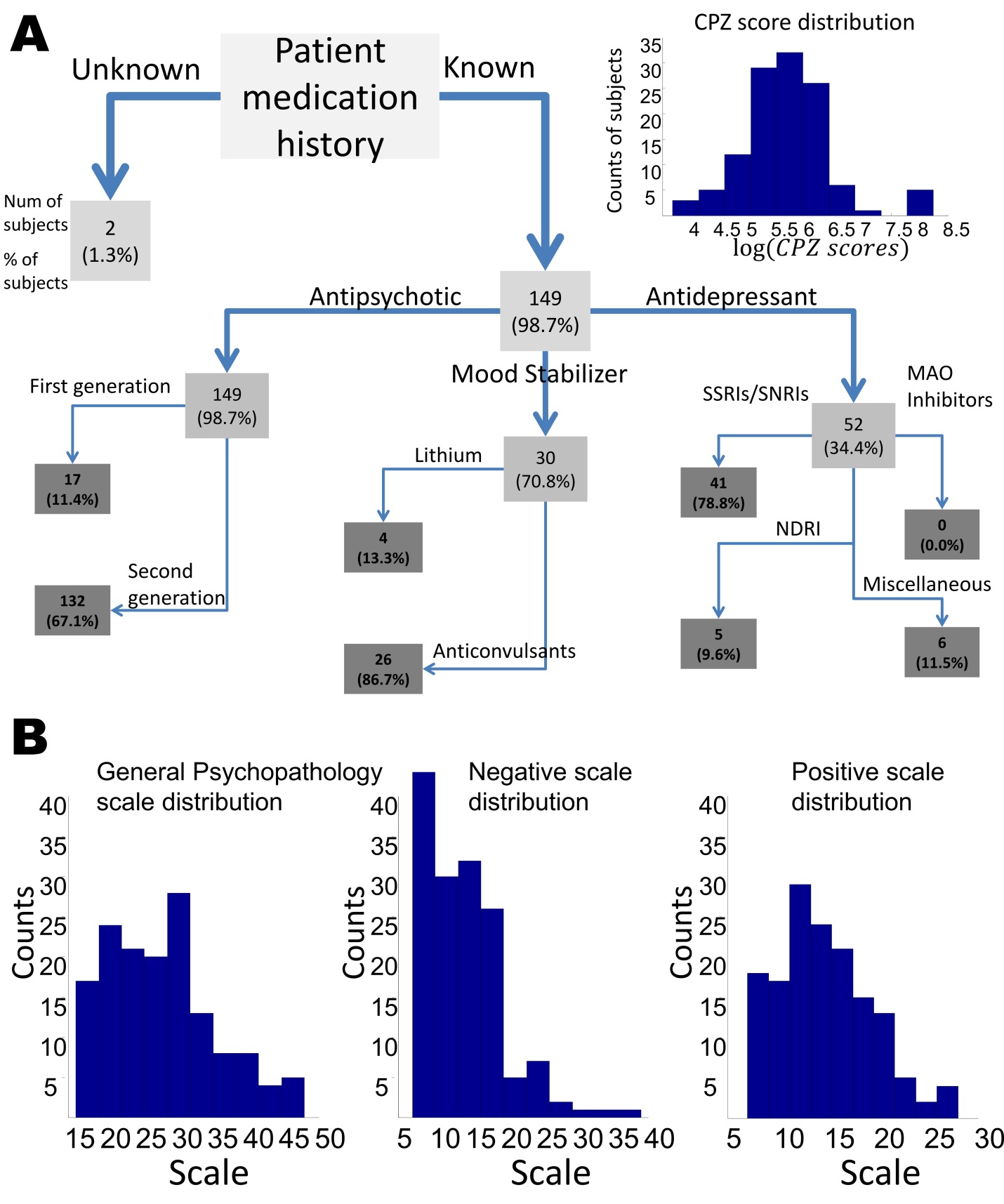


Figure S 1: (A) Graphical representation of medication information of the patients and distribution of chlorpromazine equivalent scores (top right corner). (B) Distribution of patients’ general psychopathology, negative and positive scales.

# B

### Group spatial independent component analysis and post-processing

Initially, subject-level voxel-wise data was decomposed into 100 spatially orthogonal components with corresponding coefficients using principal component analysis (PCA) followed by concatenation of this reduced data for all subject and second PCA reduction into 100 orthogonal components and the coefficients. The infomax algorithm is used to make these orthogonal components maximally independent from one another. This is followed by a spatio-temporal back-reconstruction step to estimate subject-level spatial maps and time-courses. These independent components and the corresponding time-courses are further analyzed to remove components overlapping with white-matter, known vascular and ventricular as well as components whose spatial maps overlaps the edge of the brain which is hypothesized to be susceptible to the motion as well as those with small low/high temporal frequency. 47 components were selected as the intrinsic functional networks (ICN) subsequently referred to as brain networks.


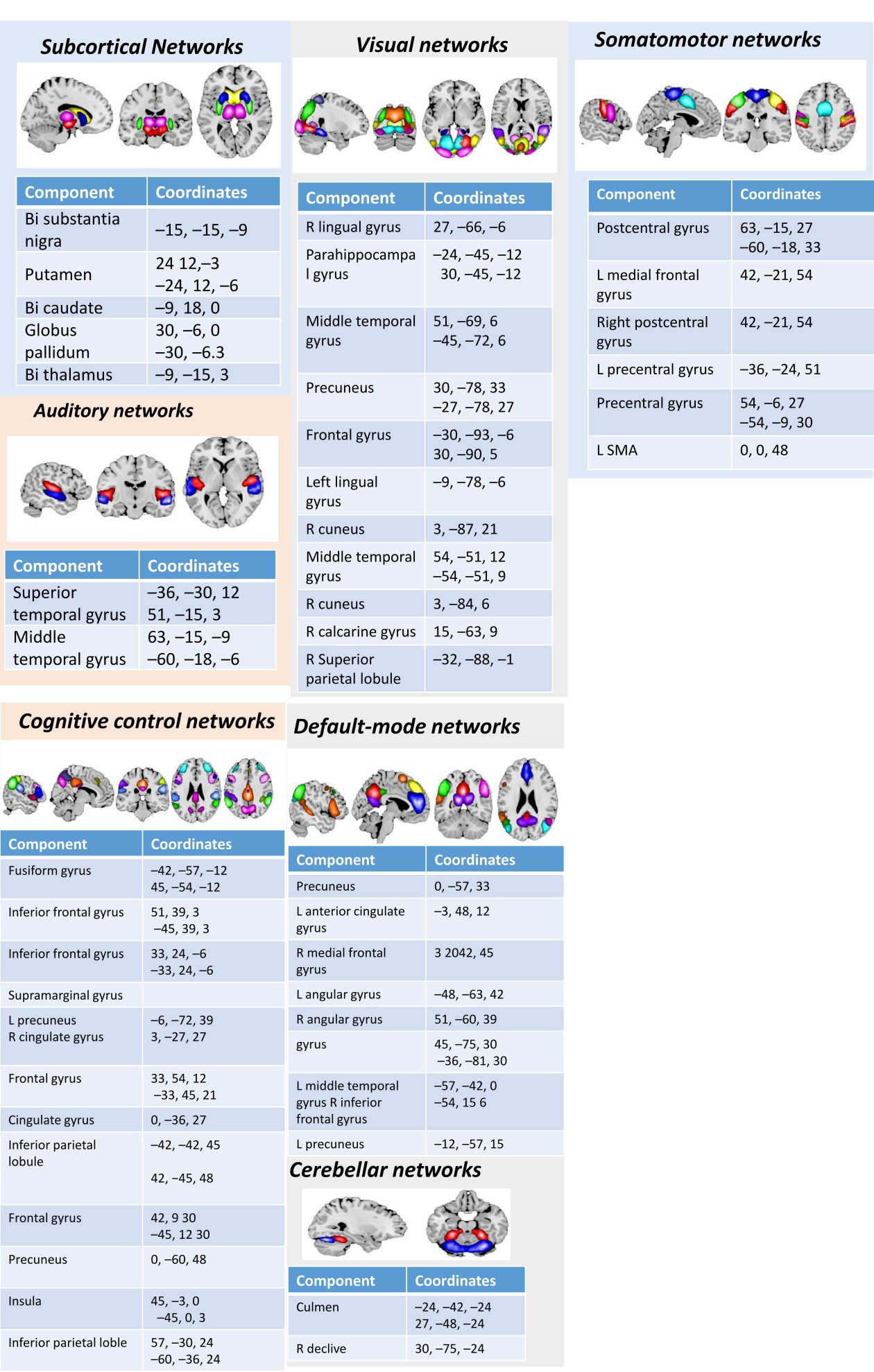


Figure S 2: ICA components identified as intrinsic functional networks of the brain.

# C

### Contrast analysis of dynamic coherence between HC and SZ states

To see contrast of HC and SZ states between maximally correlated states (set 1 of the results) or same states (set 2) with respect to dynamic coherence, for each pair of components and each pair of co-occurring states, we first derive the distributions of both the phases^[[1]](#footnote-2)^ and amplitudes of network-pair dynamic coherences of the subjects in each group. Second, we separately test the null hypothesis that the median of these distributions are the same between the groups. The estimated p-values of the tests, are corrected for multiple comparisons by Bonferroni correction method and the significance level is set to 0.01. To remove susceptibility of the dynamic coherence to the motion as well as gender and age of the subjects prior to the above analysis, we regress out variation of amplitude and phase dynamic coherence due to the selected explanatory variables (diagnosis (0: healthy, 1: schizophrenia), age, gender (0: male, 1: female), site information and average frame-wise displacement of each subject) by first, finding the best model which describes subject-wise representation of each state by using MANCOVA analysis and backward model selection[^43^](#_ENREF_43)^,^[^47^](#_ENREF_47). The response variable was subject-wise mean of each state as multivariate variable. This analysis was performed separately for phase and amplitude of the response variable. The comparisons between the phase and amplitude of the states at network-pair-level were performed only if the diagnosis variable was among those in the final reduced model suggested by the MANCOVA analysis, and only after regressing out effect of other variables in the reduced model.

1. Note that in this study, we only want to investigate differences between absolute phases of dynamic coherence between the groups and consequently all further analysis is done on the absolute value of the phase, which also makes the analysis less complicated since the measure would not be circular anymore. [↑](#footnote-ref-2)
